# HeartBioPortal 3.0: an integrated cardiovascular genomics knowledge environment for molecular, clinical and population-scale interpretation

**DOI:** 10.64898/2026.06.28.26356792

**Published:** 2026-07-04

**Authors:** Kasra Vand, Nelson Badia, Bohdan Khomtchouk, Sarath Chandra Janga

## Abstract

Cardiovascular genomics is producing rapidly expanding genetic, molecular, phenotypic and clinical data, yet relevant evidence remains fragmented across resources and difficult to translate into actionable biological and ultimately translational knowledge. HeartBioPortal (HBP) is a browser-based cardiovascular knowledge environment that was developed to address this problem by organizing omics, variant, phenotype and clinical evidence centered around gene queries. Here we describe HBP 3.0, a major update that expands both the data architecture and interpretive interface. This update introduces DataHub, a reproducible data-engineering layer for source ingestion, standardization, variant-centered aggregation, provenance tracking and compact serving artifacts. The release integrates cardiovascular clinical practice guideline context through a graph-backed clinical knowledge layer; incorporates cardiovascular summary statistics from the Million Veteran Program and public aggregate resources; expands source-preserving population frequency, variant annotation and structural-variant; and adds gene profile, drug-discovery and protein-context layers. HBP 3.0 incorporates 594.3 million allele-frequency observations across 18.1 million rsIDs, 3.04 million exon-enriched structural-variant records, 66.9 thousand protein isoforms with 3.26 million non-exon protein feature annotations, 17,128 gene-drug records, and a clinical guideline knowledge graph with 42,895 entities and 106,304 relationships. The redesigned gene dossier view combines phenotype filtering, annotation composition, persistent selected-detail panels and exportable chart data in one workflow. HBP 3.0 is designed to help cardiovascular and eventually cardiometabolic researchers move from a genetic or genomic signal to biological knowledge and potentially clinical and therapeutic context while preserving source provenance and interpretive boundaries. Database URL: https://www.heartbioportal.com/

## Full Text Availability

The license terms selected by the author(s) for this preprint version do not permit archiving in PMC. The full text is available from the preprint server.

